# Case–control study of paternal occupational exposures and childhood bone tumours and soft-tissue sarcomas in Great Britain, 1962–2010

**DOI:** 10.1038/s41416-020-0760-7

**Published:** 2020-02-26

**Authors:** Gerald M. Kendall, Kathryn J. Bunch, Charles A. Stiller, Timothy J. Vincent, Michael F. G. Murphy

**Affiliations:** 1 Cancer Epidemiology Unit, Nuffield Department of Population Health, University of Oxford, Richard Doll Building, Old Road Campus, Oxford, OX3 7LF UK; 2 National Perinatal Epidemiology Unit, Nuffield Department of Population Health, University of Oxford, Richard Doll Building, Old Road Campus, Oxford, OX3 7LF UK; 3 National Cancer Registration and Analysis Service, Public Health England, Chancellor Court, Oxford Business Park South, Oxford, OX4 2GX UK; 4 Formerly of Childhood Cancer Research Group, University of Oxford, Oxford, UK; 5 Nuffield Department of Women’s and Reproductive Health John Radcliffe Hospital, University of Oxford, Oxford, OX3 9DU UK

**Keywords:** Cancer, Sarcoma

## Abstract

**Background:**

This nationwide study investigated associations between paternal occupational exposure and childhood bone tumours and soft- tissue sarcomas.

**Methods:**

The UK National Registry of Childhood Tumours provided cases of childhood sarcomas born and diagnosed in Great Britain, 1962–2010. Control births, unaffected by childhood cancer, were matched on sex, birth period and birth registration sub-district. Fathers’ occupations were assigned to one or more of 33 exposure groups and coded for occupational social class.

**Results:**

We analysed 5,369 childhood sarcoma cases and 5380 controls. Total bone tumours, total soft-tissue sarcomas and the subgroups osteosarcoma, rhabdomyosarcoma and Ewing Sarcoma Family of Tumours (ESFT) were considered separately. Significant positive associations were seen between rhabdomyosarcoma and paternal exposure to EMFs (odds ratio = 1.67, CI = 1.22–2.28) and also for ESFT and textile dust (1.93, 1.01–3.63). There were putative protective effects on total bone tumours of paternal dermal exposure to hydrocarbons, metal, metal working or oil mists.

**Conclusions:**

Despite the large size and freedom from bias of this study, our results should be interpreted with caution. Many significance tests were undertaken, and chance findings are to be expected. Nevertheless, our finding of associations between ESFT and paternal exposure to textile dust may support related suggestions in the literature.

## Background

Childhood cancers are rare diseases, and sarcomas account for a little over 10% of them.^1^ Nevertheless, survival at 10 years is not much more than 50%,^1^ and they are diseases of public concern. The causes of childhood sarcomas are very incompletely understood, but as with other childhood cancers, paternal occupational exposures have been mooted as a possible cause.^2–5^ In this paper, we report a case–control study to investigate paternal occupational exposures as an aetiological factor in childhood sarcomas using data from the UK National Registry of Childhood Tumours. In this introduction, we give more background information about our data sources, and about the diseases under study. In particular, we outline what is known about their aetiology. Since this is unknown for most cases, interest in aetiological factors remains high. In this context, there is particular relevance in previous studies of the role of parental (particularly paternal) exposures. Any reported positive associations provide prior hypotheses for testing in this or future studies.

The UK National Registry of Childhood Tumours (UK NRCT) holds a substantially complete record of childhood cancers diagnosed in Great Britain 1962–2010 together with birth registrations for these case children and matched controls.^6^ Several papers based on these data have been published, investigating associations between inferred paternal occupational exposures to potential risk factors and individual cancer subtypes (retinoblastoma,^7^ Wilms tumour,^8^ neuroblastoma,^9^ leukaemia,^10^ central nervous system tumours^11^ and lymphomas^12^). This paper concludes the series by investigating possible associations of paternal occupational exposure and childhood malignant bone tumours, and soft tissue and other extraosseous sarcomas using cases, both fatal and non-fatal, from the UK NRCT.

Malignant bone tumours and soft-tissue and other extraosseous sarcomas are defined as Groups VIII and IX, respectively, in the International Classification of Childhood Cancer version 3 (ICCC-3).^13^ The ICCC-3 codes are 81–85 and 91–95, respectively. We will refer to these collectively as “sarcomas”, though the terminology might be questioned for a small number of bone tumours (ICCC-3 84 and 85, together amounting to fewer than 2% of study subjects). Bone and soft-tissue (ST) sarcomas together account for around 11% of cancers diagnosed in children aged less than 15 years in Great Britain, Europe and North America;^1,14^ ST sarcomas typically account for 60% of this total. Bone and ST sarcomas account for a similar proportion of childhood cancer in most other world regions, with the exception of sub-Saharan Africa where they are relatively more prevalent.^14^ In recent years, the total annual numbers in the United Kingdom have been about 80 bone tumours and 120 soft-tissue sarcomas.^15^ Between 1966 and 2005, the recorded incidence increased at an annual average rate of 0.5% for bone tumours and 1.6% for ST sarcomas, compared with 1.0% for childhood cancer overall.^16^

Incidence rates for malignant bone tumours increase with age during childhood, and are similar in boys and in girls.^1,17^ The most frequent subgroups are osteosarcomas (ICCC-3 81) and Ewing Sarcoma (ICCC-3 83); other types of bone tumours, including chondrosarcoma, chordoma and fibrosarcoma, are rare in childhood. The incidence of ST sarcomas generally decreases with age. For boys, there is a sizable increase between about 3 and 8 years; otherwise the rates in both sexes are similar.^1^ The most frequent subgroups are generally rhabdomyosarcomas (ICCC-3 91), fibrosarcomas (92) and the Ewing Sarcoma Family of Tumours (94.1 and 94.2). The principal exception to this pattern concerns Kaposi sarcoma, which is one of the most frequent childhood cancers in much of sub-Saharan Africa, where prevalence of HHV8 and HIV is high,^18^ but is very rare among children elsewhere.

Ewing sarcoma was originally defined as a bone tumour, but tumours of the Ewing Sarcoma Family can also arise in many other sites, and increasing numbers of cases have been diagnosed and classified as extraosseous in origin. For this reason, it makes sense epidemiologically to consider all ESFTs together, by combining ICCC-3 83, 94.1 and 94.2.^1,19^

### Aetiology of childhood bone tumours and soft-tissue sarcomas

A comprehensive review of the aetiology of both adult and paediatric sarcomas was published by Burningham et al.^20^ In addition, Thomas and Ballinger^21^ list over 30 risk factors for sarcomas, divided into heritable, environmental and other. For two-thirds of these risk factors, the quality of the supporting evidence was judged to be high. Here, we concentrate on paediatric sarcomas, on more recently published papers and in particular on the influence of parental exposures. In so far as the distinction is practicable, we deal first with aetiological factors generally before discussing the possible role of parental exposures. Our interest is mainly in the latter, but if other known factors accounted for at least a majority of sarcomas, there would be less reason to consider parental exposures as of potential importance.

Several aetiological factors have been implicated in childhood sarcomas. Birthweight has often been reported to play a role with higher risks to heavier infants. In an early study, Hartley et al.^22^ reported lower birthweight in children with Ewing’s tumour. Bjørge et al.^23^ reported that newborns large for gestational age were at increased risk of connective/soft-tissue tumours. In a UK/US study of 40,000 cases of childhood cancer, O’Neill et al.^24^ reported a strong association between birthweight and risk of ST sarcomas, but no such association was found for bone tumours. However, recent publications have not found persuasive evidence of such an association.^25–27^

A number of genetic conditions are linked with these diseases.^20,28^ Lupo et al.^29^ found that having a congenital malformation was associated with STS. Childhood osteosarcoma appears to be more common when bone is developing rapidly, in particular around the time of puberty.^30^ Ionising radiation is an established cause of both bone and soft-tissue sarcomas, as a result of both external and of internal radiation.^31–35^ However, it should be noted that this evidence largely involves sarcomas diagnosed after childhood. Nevertheless, Magnani^36^ reported a non-significantly higher rate of in utero diagnostic radiology in mothers of children who developed soft-tissue sarcomas compared with controls (OR = 1.9, CI = 0.5–6.5).

Fluoride is sometimes added to drinking water in order to help reduce dental caries. It has been suggested that this leads to elevated levels of bone cancers.^37^ However, there has been no clear demonstration of such a risk.^38^

Despite the multiplicity of putative risk factors, it should not be concluded that the origins of the majority of childhood sarcomas are well understood. This is in part because of the multiplicity of sarcoma subtypes and in part because of their rarity. Even a very large prospective study^39^ included too few childhood sarcomas for useful conclusions to be drawn.

### Occupational exposures of parents

In the present context, parental occupation and associated exposures are the group of possible aetiological factors of greatest interest. There are a number of studies in the literature, and several possible aetiological factors have been suggested. These are of particular relevance since they provide prior hypotheses for this work. However, sarcomas are rare, and this limits the evidence available. Thus, Savitz and Chen^40^ in a general study of parental occupation and childhood cancer reported nothing about childhood sarcomas. Nor did Colt and Blair,^2^ in an update of this study.

Hum et al.^41^ reported that the risk of Ewing sarcoma was significantly elevated among children whose fathers worked in the social sciences, and a greater risk of Ewing sarcoma in mothers who were teachers. The authors noted that a number of studies had identified white-collar parental occupations as being associated with increased risk, even though hazardous exposures are not expected to occur in these occupations. Hum et al. suggested that these findings were influenced by socioeconomic status, but did not have data to test the hypothesis. Olsen et al.^42^ reported statistically significant associations between childhood bone tumours and parental employment in education, health and welfare, and between soft-tissue sarcomas and paternal occupation in iron or shipyards or municipal administration. Maternal occupation in medical and dental services was associated with both bone and soft-tissue sarcomas. However, Olsen et al. were cautious in the interpretation of their findings. Conversely, Gelberg et al.^43^ reported no significant associations between parental occupation and osteosarcoma.

Magnani et al.^36^ reported an interview-based study of 36 cases of paediatric soft-tissue sarcomas and 326 controls. A number of possible aetiological factors were investigated including parental occupation. Some positive associations with either maternal or paternal occupational histories were identified, but Magnani et al. found them difficult to interpret in view of the large number of comparisons and small absolute numbers. This caution in the interpretation of a fairly small study is wise. However, we note that Magnani et al. reported some findings of possible relevance to the present study. There were statistically significant associations between childhood soft-tissue sarcomas and prenatal maternal occupation in farming (OR = 7.0, CI = 1.5–33.2), textile weaving (OR = 4.3, CI = 1.0–18.0) and textile spinning (OR = 7.0, OR = 1.5–33.2). The association with antenatal radiology was noted above.

There is a considerable literature on associations between childhood cancers and parental involvement in farming or work with agrochemicals. A number of studies have suggested links between childhood sarcomas and the former.^41,44–46^ In particular, Valery et al.^45^ conducted pooled analyses and also meta-analyses of studies investigating links between parental occupation and farming. These authors concluded that this collaborative analysis supported the hypothesis of an association between ESFT and parental occupation in farming. The odds ratio was 2.3 (95% CI = 1.3–4.1) for children whose fathers had worked on farms during the peri-conception and gestation periods. No other occupational group had other than minor inconsistent associations with the occurrence of ESFT. However, Moore et al.^47^ suggested that reported associations with farming were in fact due to organic dusts encountered when working on a farm.

Associations between childhood cancers and pesticide exposures were reviewed by Zahm and Ward,^48^ who considered 22 studies published up to 1998. This work was updated by Infante-Rivard and Weichenthal,^49^ who considered a further 21 publications. Infante-Rivard and Weichenthal concluded that a number of epidemiological studies consistently reported increased risks between pesticide exposures and a number of childhood cancers including Ewing sarcoma. A meta-analysis of recent epidemiological studies of pesticide exposure and cancer in offspring^50^ found a strong association between childhood Ewing sarcoma and parental exposure to pesticides (OR = 2.01, 95% CI 1.45–2.79). However, the authors advised caution in the interpretation of this finding because of the small number of studies involved. In a specific study, Grufferman^51^ reported little evidence that parental exposure to Agent Orange influences the risk of rhabdomyosarcoma in offspring.

There have thus been numerous studies of the role of farm work or of agrochemicals in the aetiology of childhood cancers and of childhood sarcomas in particular. The sheer weight of studies caused Infante-Rivard and Weichenthal^49^ to conclude that “one can confidently state that there is at least some association between pesticide exposure and childhood cancer”. However, Infante-Rivard and Weichenthal also noted that most of the studies reviewed by themselves or by Zahm and Ward^48^ suffered from very imprecise exposure assessment. A particular problem with studies of farming is that it may be difficult to distinguish any effects of parental exposure from effects caused by direct exposure of their offspring.

A case/control study by Pearce et al.^52^ examined cancers in the offspring of men occupationally exposed to ionising radiation or to electromagnetic fields (EMFs). There was potential for very large overlaps between exposures to the two agents. In a total of over 4,700 cases of cancers arising up to the age of 25,246, case fathers were, on the basis of birth certificate occupation, likely to have been exposed to ionising radiation or to EMFs. Increased risks of chondrosarcoma (OR 8.7, 95% CI 1.55–49.4) were seen in the offspring of men exposed on the basis of two exposed and six unexposed fathers.

### General observations on the study

Our aim was to investigate any associations between the incidence of childhood sarcomas and paternal occupational exposures in the context of previous studies in this area. We did so, using exposures inferred from the father’s occupation as given on the birth registration details. These exposure classes were broad, generic and in no way specific to the individual concerned.^53^ Over the long period covered by this study, some important factors may have changed: diagnostic practice and occupational exposures are prime examples. These are considered in the “Discussion” section.

## Methods

### Cases and controls

In total, 6,289 registered cases of sarcomas as the first primary malignancy in children aged <15 born and diagnosed between 1962 and 2010 in Britain were identified from the UK NRCT. A total of 285 cases were excluded because they were born overseas or adopted. A further 169 cases for whom no birth registration could be found were also excluded, leaving 5,835 eligible cases for whom a birth record was available (Table 1).

**Table 1:** Numbers of case and control records in different categories for birth registrations and coding of occupation and social class.

Birth registration	Cases	Controls
Born and diagnosed in 1962–2010 in NRCT	6289	
Born overseas/adopted	285	
Late registrations—birth record not requested	6	
Not traced in birth registers	163	
Eligible and birth record available	5835	
Occupation coding	Cases	Controls
Total eligible birth registrations	5835	5835
Missing paternal occupation	378	386
Unable to classify to 1980 occupational classification	62	49
Unable to convert to 1970 classification	26	20
Total eligible for unadjusted occupational analysis (Tables B4–B8)	5369	5380
Social class coding		
Social class based upon occupation missing**	580	596
Total eligible for occupational social class analysis (Table 1)	5255	5239

**Where occupation not given, armed forces, students, independent means and permanently sick.

Control children were selected from birth registers, held by the then Office for National Statistics (ONS) or the General Register Office for Scotland (GROS). For this study, one control for each case was used, matched on sex, period of birth and birth registration sub-district.

The completeness of ascertainment of childhood cancer cases in the NRCT has varied over time, but it contains a substantially (>97%) complete record of all childhood cancers registered in Britain from the early 1970s.^1,54,55^

Oxfordshire Research Ethics Committee (Oxfordshire REC C, Ref 12/SC/0532) approved the use of these data in 2012.

### Coding of occupational groups

Paternal occupation is recorded on the public record of UK birth registrations where the father is named. Paternal occupation was abstracted verbatim from the case and control birth records as supplied by ONS and GROS.

Occupations were coded according to the 1980 Office of Population Censuses and Surveys (OPCS) Classification of Occupations.^56^ Coding was carried out independently by two coders; where they disagreed a third coded the occupation. Where the third coder agreed with one of the original coders that agreed code was assigned. Where all three coders disagreed, the occupation was coded as “uncodable”. At all stages, occupations were coded blind to the case–control status of the individuals.

For 378 cases and 386 controls, paternal occupation was missing, and these subjects were excluded from the analysis (Table 1). For some (62 cases and 49 controls), it was not possible to assign an occupation code, or it was not possible to convert the 1980 code to a 1970 code (26 cases and 20 controls). In these circumstances, the paternal occupation was coded as missing. This leaves 5,369 and 5,380 cases and controls eligible for the (unadjusted) analysis by occupational exposure (Tables 1, 2).

**Table 2:** Number of sarcoma cases and controls broken down by ICCC-3 code.

		Eligible and with birth record			Included in analysis		
		Cases	Controls	Total	Cases	Controls	Total
81	Osteosarcomas	1135	1135	2270	1041	1052	2093
82	Chondrosarcomas	42	42	84	39	41	80
83	Ewing tumour and related bone sarcomas	875	875	1750	809	816	1625
84	Other specified malignant bone tumours	67	67	134	62	64	126
85	Unspecified malignant bone tumours	42	42	84	42	38	80
91	Rhabdomyosarcomas	2093	2093	4186	1923	1925	3848
92	Fibrosarcomas, peripheral nerve sheath tumours	344	344	688	314	314	628
93	Kaposi sarcoma	5	5	10	5	4	9
94	Other specified soft-tissue sarcomas	957	957	1914	883	869	1752
95	Unspecified soft-tissue sarcomas	275	275	550	251	257	508
	Total over all codes	5835	5835	11670	5369	5380	10,749

### Coding of occupational exposure groups

This used a job-exposure matrix developed by Fear et al.,^57^ using occupational classifications from the 1970 Classification of Occupations.^58^ The 1980 classifications were converted to the 1970 scheme using bridge codes.^57,59^ The 1970 codes were then allocated to one or more of 33 broad occupational exposure groups, which had been associated with cancer or adverse reproductive outcomes in the offspring of men exposed to them. The occupational exposure groups are detailed in the results, figures and tables. The assignments of exposure groups to occupations were on the basis of specialist experience, examination of job descriptions and literature concerning occupational exposures.^57^ These job-exposure associations have been described in detail elsewhere.^57,60^ Occupations not appearing in any of the 33 groups were classified “unexposed” in all groups.

Occupations classified to one or more of the exposure groups were further defined as having either “definite” (daily contact with the agent, or contact at a high intensity) or “possible” (contact with the agent neither daily nor at high intensity) exposure in that group.^8,53^ Some exposures could be incurred in combination with others; thus, job titles could be coded to more than one occupational exposure group; for example, bus drivers appear as exposed in “exhaust fumes”, “inhaled hydrocarbons” and “social contact”. Details and discussion are given in the Supplementary Material on Exposures (Supplementary Tables A1, A2).

### Coding of occupational social class

Each 1980 occupation code was assigned to one of six social class codes from the 1980 OPCS Classification of Occupations.^61^ Of the eligible study subjects for whom a birth record was available, 580 cases and 596 controls social class was classified as “missing” because no occupation was given or the occupation falls outside the ONS social classifications (i.e. armed forces, student, independent means or sick) (Table 1). This leaves 5,255 cases and 5,239 controls for the social class analysis (Table 3).

**Table 3:** Childhood sarcoma risk by paternal occupationally defined social class.

	Total bone tumours			Total soft-tissue sarcomas			Osteosarcoma			Rhabdomyosarcoma			Ewing Sarcoma family of tumours		
Social class of father	Cases	Controls	OR^a^ (95% CI)	Cases	Controls	OR^a^ (95% CI)	Cases	Controls	OR^a^ (95% CI)	Cases	Controls	OR^a^ (95% CI)	Cases	Controls	OR^a^ (95% CI)
I	121	125	0.80 (0.52–1.24)	250	229	1.04 (0.76–1.44)	65	70	0.73 (0.38–1.38)	144	139	0.88 (0.58–1.32)	72	73	0.75 (0.44–1.27)
II	405	366	**1.32** (1.02–1.72)	720	636	1.04 (0.86–1.26)	202	189	1.14 (0.80–1.64)	394	367	0.98 (0.76–1.26)	256	205	**1.5** (1.07–2.10)
III Non Manual	249	267	0.84 (0.62–1.13)	373	393	0.92 (0.72–1.17)	129	128	0.86 (0.57–1.29)	198	232	0.77 (0.55–1.08)	132	152	0.82 (0.54–1.24)
III Manual	730	718	1.00	1220	1215	1.00	392	388	1.00	716	675	1.00	403	412	1.00
IV	326	360	0.97 (0.75–1.25)	541	577	1.06 (0.87–1.30)	165	194	0.98 (0.69–1.40)	305	338	0.84 (0.64–1.10)	183	184	1.21 (0.85–1.73)
V	121	131	0.80 (0.53–1.19)	199	222	0.88 (0.63–1.23)	71	63	0.93 (0.54–1.60)	123	125	0.98 (0.63–1.51)	64	75	0.92 (0.53–1.61)
Trend analysis^b^	1,952	1967	0.96 (0.91–1.01)	3303	3272	**0.96** (0.92–1.00)	1024	1032	0.98 (0.91–1.05)	1880	1876	0.98 (0.93–1.03)	1110	1,101	0.95 (0.89–1.02)

The table includes 26 cases and 20 controls who had a social class code assigned, but who had no occupation code assigned, and were excluded from the occupation analysis.

ORs in bold are significant *P* < 0.05.

^a^OR for the indicated ONS Social Class(es) with III Manual taken as the reference category.

^b^OR for each increase in occupational social class.

### Analysis

Odds ratios (ORs) and 95% confidence intervals (95% CIs) were calculated using conditional logistic regression.^62^ Cases and controls were individually matched on sex, period of birth and birth registration sub-district; thus, the main analyses include implicit adjustment for these variables. ORs and 95% CIs additionally adjusted for social class (I, II, IIINM, IIIM, IV and V) were also generated. For brevity, these will be referred to as the “unadjusted” and “adjusted” analyses, respectively.

Our primary exposed population was those classified as “definitely” exposed. The same analyses were repeated taking the exposed population as those with either “definite” or “possible” exposures. Differences between these two sets of results were generally small, and we attach the greatest weight to definite exposures. Where appropriate, the results from the “Definite and possible” analyses are noted below.

All statistical analyses were carried out using STATA 15,^57^ usually with the Clogit command. Clogit gives unreliable results if the number of cases/controls is small, and if there were five or fewer exposed cases and/or controls for any analysis, the exact conditional logistic regression was used (the Stata Exlogistic command). Due to the small numbers of cases/controls, adjustment for social class was inappropriate in these cases. Statistically significant results were defined as those where the *p*-value was < 0.05.

The results are shown as forest plots in the main text. In these figures, data for exposures with fewer than five cases and/or controls have been suppressed. However, full numerical results are given in Supplementary Material on Detailed Results.

## Results

As described above, Table 1 gives details of the records excluded at various stages of setting up the set of cases and controls for analyses. After exclusions, a total of 5369 (92%) cases and 5380 (92%) controls were included in the unadjusted analyses of occupation and sarcoma risk, and 5255 (90%) of cases and 5239 (90%) of controls in analyses of social class and sarcoma risk. Supplementary Table B1 gives breakdowns of the eligible study population by demographic factors. There was little difference between the cases and controls in these factors, which included social class.

Table 2 gives breakdowns by ICCC-3 code of both the eligible population and those included in the occupational exposure analysis. It can be seen that the excluded records are not concentrated in any particular area. Table 2 shows that of the included study subjects, 4,004 (37%) had malignant bone tumours and 6,745 (63%) soft-tissue and other extraosseous sarcomas. Within the bone tumours 2093 (52%) were osteosarcomas. Within the soft-tissue sarcomas 3,848 (57%) were rhabdomyosarcomas. There were 2214 tumours belonging to the Ewing Sarcoma family of tumours, 21% of all tumours.

The 5369 cases had occupations with which a total of 6097 exposures were associated (Supplementary Table A1). The number of exposures per case varied from zero to five (Supplementary Table A1). The pattern for controls was broadly similar. About 36% of cases and controls had occupations with which none of the selected exposures were associated, and about 34% had a single exposure; the remaining 30% had between two and five exposures. Details are given in Supplementary Table A2.

**Fig. 1:**
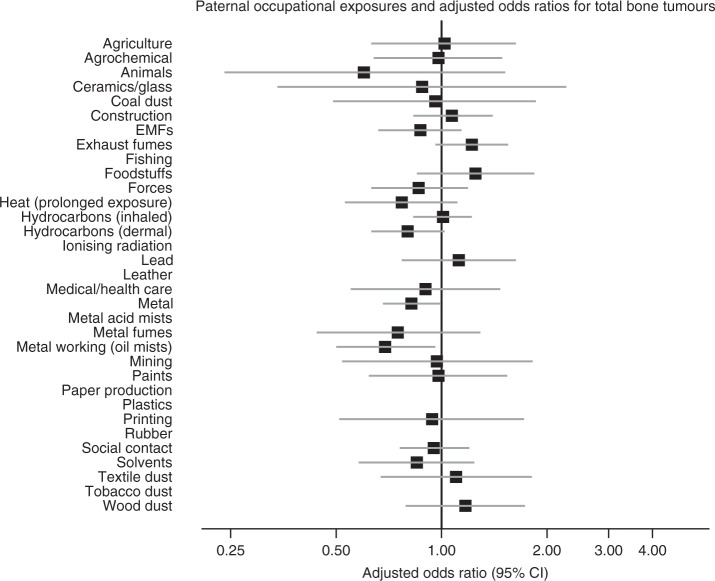
Paternal occupational exposures and total risk of childhood bone tumours.

Figure 1 and Supplementary Table B2 show estimates for the risk of total bone tumours by the occupational exposure group. In the unadjusted analysis, bone tumour risk was significantly reduced at the 5% level in the children of fathers exposed to dermal hydrocarbons (OR = 0.76, 95% CI = 0.60–0.97), to metal (0.79, 0.66–0.95) and to oil mists during metal working (0.66, 0.48–0.90). In the last of these, the reduction was significant at the 1% level. The reductions were also significant in analyses adjusting for occupational social class, except for dermal exposure to hydrocarbons. It should be noted that no father was exposed to metal oil mists who was not also exposed to dermal hydrocarbons. The reductions are slightly smaller in the analysis adjusted for social class, and that for dermal exposure to hydrocarbons is no longer significant.

**Fig. 2:**
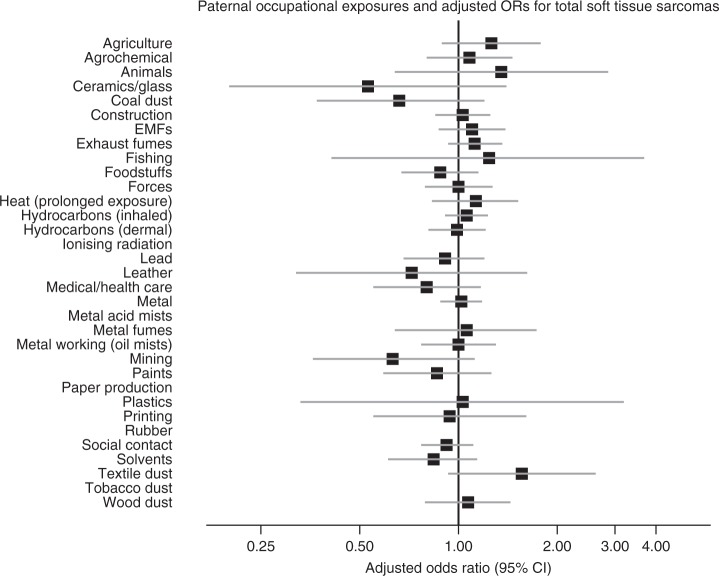
Paternal occupational exposures and total risk of childhood soft-tissue sarcomas. Paternal occupational exposures and total risk of childhood soft-tissue sarcomas.

Figure 2 and Supplementary Table B3 show estimates for the risk of total soft-tissue sarcomas by the occupational exposure group. No odds ratios were significantly different from 1 with or without adjustment for occupational social class when considering definite exposures. When considering definite and possible exposures, the risk for exposure to textile dust was similar to that for definite exposure, but increased numbers resulted in a significantly raised adjusted odds ratio (1.54, 1.01–2.36, *p* < 0.05; result not shown in tables).

**Fig. 3:**
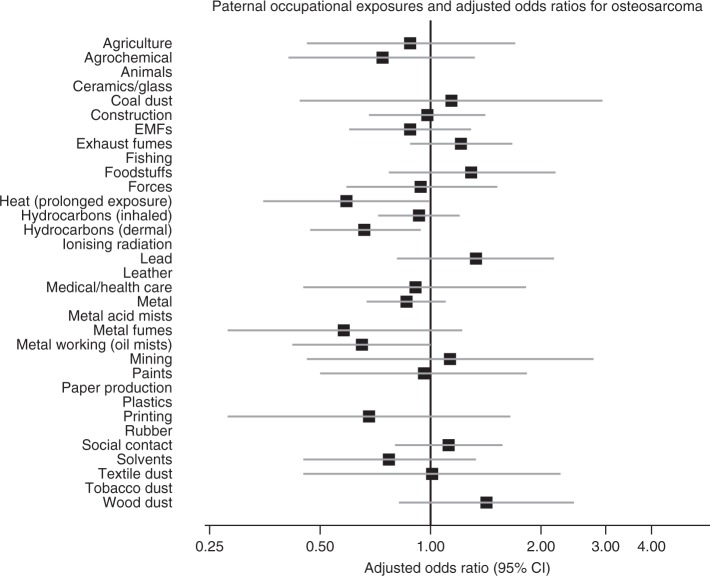
Paternal occupational exposures and risk of childhood osteosarcoma.

**Fig. 4:**
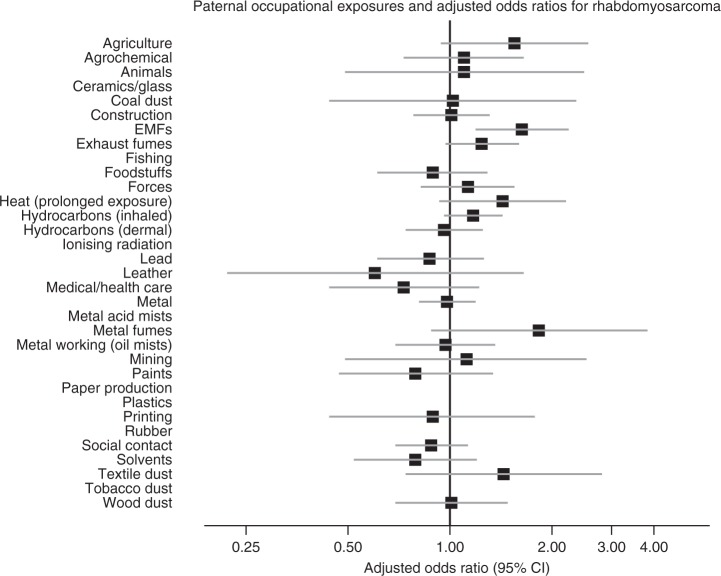
Paternal occupational exposures and risk of childhood rhabdomyosarcomas.

**Fig. 5:**
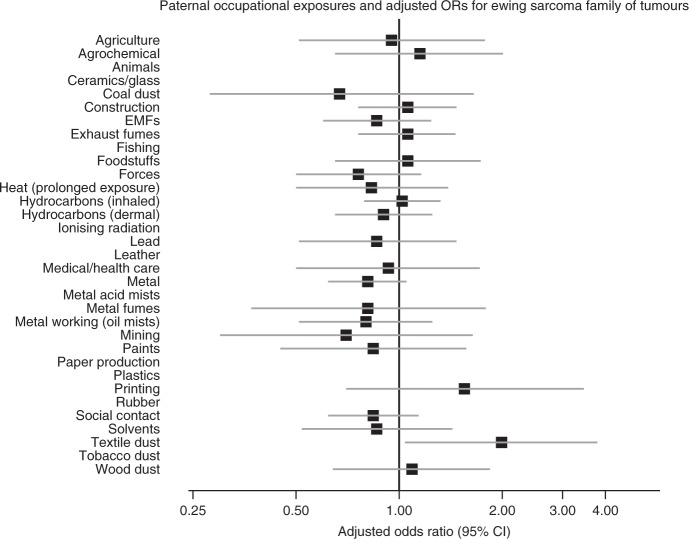
Paternal occupational exposures and risk of childhood Ewings Family tumours.

Figures 3–5 (Supplementary Tables B4, B5, B6) present data for the major sarcoma subgroups separately. In analyses unadjusted for social class and osteosarcomas, odds ratios are significantly reduced for exposures to prolonged heat (0.58, 0.34–0.96), to dermal exposure to hydrocarbons (0.64, 0.46–0.89) and to metal-working oil mists (0.62, 0.40–0.95). For dermal exposure to hydrocarbons the reduction was significant at the 1% level. Adjustment for occupational social class resulted in only slight increases in reduced risk.

For rhabdomyosarcomas, odds ratios were significantly elevated in the children of men exposed to electromagnetic fields (1.67, (1.22–2.28)). After adjustment for occupational social class the OR became 1.63 (1.19–2.24). The results were similar but less marked when both definite and possible exposures were considered with minimal change on adjustment (unadjusted: OR = 1.31, 1.02–1.68, *p* < 0.05; adjusted: OR = 1.31, 1.01–1.69, *p* < 0.05; results not shown in tables). Embryonal rhabdomyosarcomas form a histologically distinct group.^1^ We found no evidence that the association depended on whether the rhabdomyosarcoma was embryonal or otherwise. Fifty-nine percent of the rhabdomyosarcomas were embryonal (i.e. of ICDO3 types 89103 or 89913). For this subset, the unadjusted OR is 1.53 (1.00–2.33), *p* = 0.048; after adjustment for social class the OR is 1.49 (0.97–2.28), *p* = 0.07.

For ESFT, odds ratios were significantly elevated for the children of men exposed to textile dust (1.93, 1.01–3.68, *p* < 0.05). There was little change on adjustment for SES, but the odds ratios became somewhat larger and more significant when both definite and possible exposures were considered (unadjusted: OR = 2.22, 1.27–3.88, *p* < 0.01; adjusted: OR = 2.23, 1.27–3.90, *p* < 0.01; results not shown in tables).

Table 3 gives a breakdown of sarcoma risk by occupational social class. As with the exposure analysis, the results are presented for total bone tumours, total soft-tissue sarcomas and the subgroups osteosarcoma, rhabdomyosarcoma and ESFT. ORs were calculated relative to Class III Manual. Odds ratios for trend were all below unity (suggesting higher risks in more affluent families), but only for all soft-tissue sarcomas taken together did such a trend become statistically significant.

## Discussion

The exposures most consistently associated with childhood sarcomas in previous studies were perhaps agriculture and agrochemicals (Exposure groups 1 and 2).^41,44–46^ We found no such associations. In no case were odds ratios in the present study significantly different from one for these exposure groups, and this study, subject to the caveats below, thus provides no support for the suggestions in the literature. Pesticides have been particularly suggested as a risk factor, but we are unable to distinguish them from agrochemicals in general.

Ionising radiation is a known cause of sarcomas, but no associations were found in this study. This is unsurprising in view of the low numbers of exposed subjects and the low doses probably incurred.

We found an association between EMFs and rhabdomyosarcoma, but no excess was apparent on soft-tissue sarcomas as a whole. Chance must be a possible explanation for our finding. In view of a report in the literature,^52^ we undertook a specific analysis of associations between EMFs and chondrosarcoma. There were only 61 chondrosarcomas in the set of eligible cases with birth records (42 with ICCC-3 82 and 19 with ICCC-3 94, division 9). No association was found (OR = 1.00, 0.07–13.80, *p* = 1.00). The OR and p value were unchanged if analysis was limited to the records with ICCC-3 82. These results are not shown in the tables.

We found an elevated risk of ESFT for the children of men exposed to textile dust. This finding was reproduced in sub-analyses. As described in the introduction, an association between childhood soft-tissue sarcomas and maternal work in the textile industry was found by Magnani et al.^36^ We also note that Moore et al.^47^ reported that the risk of Ewing sarcoma was increased with probable parental exposure to wood dusts, and suggested that this might be extended to organic dusts more generally. Of course, not all textile dusts are “organic” in this sense, though it is likely that most dusty textile operations do indeed involve natural fibres such as cotton or wool.^63^ Nevertheless, the possibility that our finding is due to chance cannot be excluded.

No associations were found for healthcare workers for whom a previous investigation had reported an association.^42^

We have investigated associations between childhood sarcomas and paternal exposures to over 30 agents. In the absence of specific prior hypotheses potential problems of multiple significance testing inevitably arise. Any significant ORs reflecting associations not previously reported in the literature were re-assessed using the Bonferroni method.^64^ None of the results above reached statistical significance using this test. In these circumstances, simple p values are likely to suggest significance for associations that are simply due to chance. However, the Bonferroni correction is likely to fail to identify genuinely significant associations. We suggest that further information and in particular additional independent studies are required to resolve such ambiguities.

As well as positive associations (increased risks in the offspring of exposed fathers) we found a number of negative associations. In particular, bone tumour risk was significantly reduced in the children of fathers exposed to dermal hydrocarbons, to metal or to oil mists during metal working. Of course, the play of chance is as likely to throw up false-negative associations (“protective effects”) as false positives (“causal associations”). However, on biological grounds, while the finding of a positive association prompts the idea that there is perhaps a causal link, a negative association seems much less likely to point to a protective effect. Chance again seems a possible explanation for our specific findings.

### Strengths and limitations

The strengths of this study are that the analysis is based on over 5000 cases with data drawn from the UK NRCT that has, over the period studied here, consistently high levels of case ascertainment.^54^ Interview-based case–control studies are often beset with recall and participation bias. These are very unlikely to arise in the present study since routinely collected data were used, and occupation was documented before diagnosis. Exposure assessment used a well-established occupational and exposure classification,^57^ to which father’s occupation was coded blind to case–control status.

However, as noted previously,^12^ our method used self-reported occupation recorded at the time of the birth of the child. In addition, our exposure categories are based on broad generic assessments; we have no individual information on the frequency or duration of exposure. Generally speaking, individual exposure assessments are to be preferred;^65^ however, we note that in a study of lung cancer, Pannett et al.^53^ found that direct exposure estimates offered little advantage over those provided by a job-exposure matrix of the kind used here. A further point is that occupational practices and exposures may also have changed during the long study period that could lead to exposure misclassification. It would be expected that health and safety regulations and protective equipment became more effective over time. In particular, the Health and Safety at Work Act of 1974 may have been influential.

Between 1966 and 2000, the recorded incidence of Ewing sarcoma increased significantly, whereas the incidence of neuroblastoma among children aged 5–14 decreased.^1^ Historically, neuroblastoma and Ewing sarcoma were difficult to distinguish on histological grounds,^66^ and ESFT was almost certainly under-diagnosed in earlier years. While this will have slightly reduced the power of the present study to detect associations of ESFT with paternal occupation, it is improbable that the diagnostic choice will have been affected by the father’s occupation at the time of the birth of the child, and so is unlikely to have resulted in any important bias.

### Interpretation

As previously described,^12^ one possible reason why our data have not shown associations between paternal occupational exposures and sarcoma risk is exposure misclassification. Paternal occupational exposure may plausibly lead to childhood cancer at peri-conception, as a result of the effects of the exposure on germ cells, during pregnancy and after birth, when contaminants brought home from the workplace may affect the embryo or young child.^67,68^ We have no information about paternal occupation before or after a child’s birth was registered; the occupation (and hence exposure) may have differed, and exposure misclassification may have arisen as a result. However, this applies equally to cases and controls. Nor do we have direct information about the intensity or frequency of exposure within groups, and over the almost 50 years for which we have data, actual exposures may have changed within exposure groups as a result of changing workplace practices.

In this study, exposures were inferred from paternal occupational exposure as recorded on the birth certificate. For about 10% of cases and controls, paternal occupation was not given or could not be interpreted. It is reasonable to ask whether this might have affected our analysis. In a study of a very similar cohort (but including all types of childhood tumour), Kendall et al.^69^ compared social class derived from paternal occupation (as used here) with the Carstairs index of social deprivation^70^ derived from data for the census ward of birth. The latter was available for all study subjects. Fathers who did not specify their occupation tended to come from more deprived areas than those who did (53% of those who did not specify occupation were from the most deprived quintile vs. 34% of those who did). These men almost certainly had a different pattern of occupations from those who gave up their occupation. However, it is highly implausible that any difference between cases and controls could arise since it is extremely rare for there to be any indication that a child has or will develop cancer at the time of birth.

## Summary

We conducted a large nationwide case–control study of childhood sarcoma and paternal occupational exposure to potential carcinogens. The study was record-based, and participation and recall bias are unlikely. Paternal occupational exposure to electromagnetic fields was associated with childhood rhabdomyosarcomas. We found no support for an equivalent association previously reported for chondrosarcoma. We found an elevated risk of ESFT in offspring of men exposed to textile dusts. It may be relevant that other studies have reported associations with wood and perhaps with organic dusts more generally. However, the possible role of chance must not be forgotten, particularly in view of the number of significance tests that we have applied. Chance is likely to account, for example, for the negative associations that we report.

Perhaps, the main weaknesses of the study are that the exposures are based on self-reported paternal occupation at the time of the child’s birth, which may or may not be the most aetiologically important period; the assignment of exposures was generic and unchanging over the study period.

## Supplementary information

Supplementary Material on Exposures and Detailed Results

## Data Availability

The data are contained within the National Registry of Childhood Tumours.
