# Supplementary material for: Case–control study of paternal occupational exposures and childhood bone tumours and soft-tissue sarcomas in Great Britain, 1962–2010
Source: Br J Cancer. 2020 Feb 26;122(8):1250–9. doi: 10.1038/s41416-020-0760-7 (PMC7156690; doi:10.1038/s41416-020-0760-7)
Supplement: Supplementary file 1 — Supplementary Material on Exposures and Detailed Results [file 41416_2020_760_MOESM1_ESM.doc]

**Supplementary Material on Exposures and Detailed Results**

Supplementary Information on Exposures

Supplementary Table A1 - Number of exposures per study subject

Supplementary Table A2 - Number of exposures to each of the agents by Case-Control status

Supplementary Information on Detailed Results

Supplementary Table B1 - Breakdown of Bone and ST Sarcoma Cases and Controls by demographic factors

Supplementary Table B2 - Paternal Occupational Exposures and ORs for total Bone Tumours

Supplementary Table B3 - Paternal Occupational Exposures and ORs for total Soft Tissue Sarcoma

Supplementary Table B4 - Paternal Occupational Exposures and ORs for Osteosarcoma

Supplementary Table B5 - Paternal Occupational Exposures and ORs for Rhabdomyosarcoma

Supplementary Table B6 - Paternal Occupational Exposures and ORs for Ewing Sarcoma Family of Tumours

**Supplementary Material on Exposures to Potentially Hazardous Agents in Specific occupations**

We determined the occupations of our cases and of controls from the paternal occupation as shown on the birth certificate of the child in question. We chose to study paternal occupations because, for our study period (1962-2010) the corresponding data for mothers were very much less complete.

As described in the main text, exposure groups were inferred using a job-exposure matrix developed by Fear et al using occupational classifications from the 1970 Classification of Occupations (which includes around 220 occupational classifications). The 1980 classifications (of which there were over 350) were converted to the 1970 scheme using bridge codes as described in the main text. The 1970 codes were then allocated to one or more of 33 occupational exposure groups, which had been associated with cancer or adverse reproductive outcomes in the offspring of men exposed to them. Inevitably, given the nature of the exercise, these exposure groups are fairly broad. Thus, there is a single category “EMFs” which covers a variety of exposure types. Occupations likely to be associated with these exposures have been identified in published literature [1, 2]. The 33 occupational exposure groups are listed in the results figures and tables. The occupations classified to each of them are detailed in Table 2 of Fear et al 1999[1].

In this Supplementary Material we provide extra detail of these occupational exposures too bulky for the main text. In what follows the reader should remember that the father’s occupation determines the agents to which he was (likely to be) exposed.

Table A1 shows the exposures associated with the various categories of employment of the fathers of the study subjects. There were between 0 and 5 exposures per occupation and the relative numbers of exposures for controls were similar to those for cases. Just over a third of study subjects had fathers in occupations associated with none of the selected agents and roughly the same proportion with a single agent. The remainder were likely to be exposed to two or more (up to five) agents

The occupations with 5 exposures were

19 Rolling, tube mill operators, metal drawers (4 controls)

41 Motor mechanics, auto engineers (79 controls; 85 cases)

85 Compositors (6 controls; 10 cases)

Exposures to a given agent might occur in more than one occupation. Thus “Social Contact” was likely in 36 occupations and “Inhaled hydrocarbons” in 30.

Table A2 shows the number of cases and controls likely to be exposed to each of the agents under study. Because some occupations are associated with exposures to more than one agent, in many cases these exposures occurred in combination rather than singly. Table A2 gives, for cases and controls together, the numbers of exposures incurred on their own and the number incurred in combination. It is clear that such exposures to more than one agent must be borne in mind when interpreting any positive finding. This is discussed in the main text.

References

1. Fear N, Roman E, Reeves G*, et al.* Father's occupation and childhood mortality: analysis of routinely collected data. Health statistics quarterly 1999;2:7-15.

2. Fear NT, Roman E, Reeves G*, et al.* Are the children of fathers whose jobs involve contact with many people at an increased risk of leukaemia? Occup Environ Med 1999;56(7):438-442.

Associated Tables:

Table A1: Number of exposures per study subject

Table A2: Number of exposures to each of the agents by Case/Control status.

Table A1: Number of exposures per study subject

|  | Cases | | | Controls | | | Total | | |
| --- | --- | --- | --- | --- | --- | --- | --- | --- | --- |
| Number of Exposures | Frequency | Percent | Total exposures | Frequency | Percent | Total exposures | Frequency | Percent | Total exposures |
| 0 | 1982 | 36.9 | 0 | 1937 | 36.0 | 0 | 3919 | 36.5 | 0 |
| 1 | 1806 | 33.6 | 1806 | 1809 | 33.6 | 1809 | 3615 | 33.6 | 3615 |
| 2 | 942 | 17.5 | 1884 | 960 | 17.8 | 1920 | 1902 | 17.7 | 3804 |
| 3 | 252 | 4.7 | 756 | 221 | 4.1 | 663 | 473 | 4.4 | 1419 |
| 4 | 284 | 5.3 | 1136 | 354 | 6.6 | 1416 | 638 | 5.9 | 2552 |
| 5 | 103 | 1.9 | 515 | 99 | 1.8 | 495 | 202 | 1.9 | 1010 |
| Total | 5369 | 100.0 | 6097 | 5380 | 100.0 | 6303 | 10749 | 100.0 | 12400 |
| Mean |  |  | 1.14 |  |  | 1.17 |  |  | 1.15 |

Table A2: Number of exposures to each of the agents by Case/Control status.

Also given are the number of exposures associated with no other and with some other agents ("Single" and "Multiple" respectively)

|  | ***Exposure*** | ***Cases*** | ***Controls*** | ***Total*** | ***Single*** | ***Multiple*** |
| --- | --- | --- | --- | --- | --- | --- |
| 1 | Agriculture | 120 | 107 | 227 | 0 | 227 |
| 2 | Agrochemical | 153 | 151 | 304 | 77 | 227 |
| 3 | Animals | 31 | 28 | 59 | 0 | 59 |
| 4 | Ceramics/glass | 14 | 22 | 36 | 30 | 6 |
| 5 | Coal dust | 43 | 51 | 94 | 0 | 94 |
| 6 | Construction | 402 | 409 | 811 | 811 | 0 |
| 7 | EMFs | 299 | 302 | 601 | 472 | 129 |
| 8 | Exhaust fumes | 472 | 414 | 886 | 14 | 872 |
| 9 | Fishing | 10 | 9 | 19 | 19 | 0 |
| 10 | Foodstuffs | 189 | 189 | 378 | 127 | 251 |
| 11 | Forces | 233 | 250 | 483 | 222 | 261 |
| 12 | Heat (prolonged exposure) | 162 | 176 | 338 | 0 | 338 |
| 13 | Hydrocarbons (inhaled) | 884 | 874 | 1758 | 16 | 1742 |
| 14 | Hydrocarbons (dermal) | 424 | 480 | 904 | 66 | 838 |
| 15 | Ionising radiation | 4 | 5 | 9 | 6 | 3 |
| 16 | Lead | 172 | 179 | 351 | 0 | 351 |
| 17 | Leather | 15 | 19 | 34 | 30 | 4 |
| 18 | Medical/health care | 95 | 105 | 200 | 12 | 188 |
| 19 | Metal | 798 | 851 | 1649 | 676 | 973 |
| 20 | Metal acid mists | 4 | 6 | 10 | 0 | 10 |
| 21 | Metal fumes | 65 | 82 | 147 | 0 | 147 |
| 22 | Metal working (oil mists) | 208 | 251 | 459 | 0 | 459 |
| 23 | Mining | 48 | 56 | 104 | 10 | 94 |
| 24 | Paints | 99 | 119 | 218 | 0 | 218 |
| 25 | Paper production | 2 | 3 | 5 | 5 | 0 |
| 26 | Plastics | 7 | 11 | 18 | 18 | 0 |
| 27 | Printing | 51 | 55 | 106 | 0 | 106 |
| 28 | Rubber | 9 | 9 | 18 | 0 | 18 |
| 29 | Social contact | 676 | 671 | 1347 | 524 | 823 |
| 30 | Solvents | 161 | 186 | 347 | 0 | 347 |
| 31 | Textile dust | 79 | 67 | 146 | 146 | 0 |
| 32 | Tobacco dust | 0 | 1 | 1 | 1 | 0 |
| 33 | Wood dust | 168 | 165 | 333 | 333 | 0 |
| 34 | Total | 6097 | 6303 | 12400 | 3615 | 8785 |

**Supplementary Material on Detailed Results**

As outlined in the main text, cases were from the National Registry of Childhood Tumours. Matched controls had been selected for cases previously. The breakdown of the potential study population by demographic factors is given in Table B1.

For our matched analysis Odds Ratios (ORs) and 95% confidence intervals (95% CIs) were calculated using conditional logistic regression implemented in Stata. Where the numbers of exposed cases and controls permitted the Stata Clogit command was used. Matching factors were: sex, period of birth and birth registration sub-district. ORs and 95% CIs additionally adjusted for social class (I, II, IIINM, IIIM, IV and V) were also generated. For brevity, these will be referred to as “unadjusted” and “adjusted” analyses respectively. Our primary exposed population was those classified as ‘definitely’ exposed. The same analyses were repeated taking the exposed population as those with either ‘definite’ or ‘possible’ exposures. Differences between these two sets of results were minimal and all further references are to definite exposures.

In instances where there were 5 or fewer exposed cases and/or controls for any analysis, the Stata Exlogistic command was used to perform exact conditional logistic regression. Because of the small numbers involved in these analyses, it was inappropriate to adjust for social class.

Statistically significant results were defined as those where the p value was <0.05 and any significant ORs reflecting associations not previously reported in the literature were re-assessed using the Bonferroni method to allow judgements to be made on the importance of multiple significance testing. In these circumstances, simple p values are likely to suggest significance for associations that are simply due to chance. However the Bonferroni correction is likely to fail to identify genuinely significant associations. We suggest that further information and in particular additional independent studies are required to resolve such ambiguities.

Associated Tables:

Table B1: Breakdown of Bone and ST Sarcoma Cases and Controls by demographic factors

Table B2: Numbers exposed and ORs (95% CIs) for Total Bone Tumours (ICCC3 81 to 85) by occupational exposure group

Table B3: Numbers exposed and ORs (95% CIs) for Total Soft Tissue Sarcomas (ICCC3 91 to 95) by occupational exposure group

Table B4: Numbers exposed and ORs (95% CIs) for Osteosarcoma (ICCC3 81) by occupational exposure group

Table B5: Numbers exposed and ORs (95% CIs) for Rhabdomyosarcoma (ICCC3 91) by occupational exposure group

Table B6: Numbers exposed and ORs (95% CIs) for Ewing Sarcoma Family of Tumours (ICCC3 83 divs 1&2 and ICCC3 94 divs 1 & 2) by occupational exposure group

Table B1 Breakdown of Bone and Soft Tissue Sarcoma Cases/ Controls by demographic factors

|  | Bone tumours (ICCC3 81-85) | | | | Soft Tissue Sarcomas (ICCC3 91-95) | | | |
| --- | --- | --- | --- | --- | --- | --- | --- | --- |
|  | Cases | | Controls | | Cases | | Controls | |
|  | No. | % | No. | % | No. | % | No. | % |
| **Sex** |  |  |  |  |  |  |  |  |
| Males | 1097 | 51 | 1097 | 51 | 2074 | 56 | 2074 | 56 |
| Females | 1064 | 49 | 1064 | 49 | 1600 | 44 | 1600 | 44 |
| Total | 2161 | 100 | 2161 | 100 | 3674 | 100 | 3674 | 100 |
|  |  |  |  |  |  |  |  |  |
| **Birth year** |  |  |  |  |  |  |  |  |
| 1962-1969 | 497 | 23 | 498 | 23 | 618 | 17 | 619 | 17 |
| 1970-1979 | 575 | 27 | 574 | 27 | 769 | 21 | 771 | 21 |
| 1980-1989 | 507 | 23 | 507 | 23 | 935 | 25 | 928 | 25 |
| 1990-1999 | 510 | 24 | 509 | 24 | 954 | 26 | 959 | 26 |
| 2000-2010 | 72 | 3 | 73 | 3 | 398 | 11 | 397 | 11 |
| Total | 2161 | 100 | 2161 | 100 | 3674 | 100 | 3674 | 100 |
|  |  |  |  |  |  |  |  |  |
| **Occupational Social class** |  |  |  |  |  |  |  |  |
| I | 121 | 6 | 125 | 6 | 250 | 7 | 229 | 6 |
| II | 405 | 19 | 366 | 17 | 720 | 20 | 636 | 17 |
| IIINM | 249 | 12 | 267 | 12 | 373 | 10 | 393 | 11 |
| IIIM | 730 | 34 | 718 | 33 | 1220 | 33 | 1215 | 33 |
| IV | 326 | 15 | 360 | 17 | 541 | 15 | 577 | 16 |
| V | 121 | 6 | 131 | 6 | 199 | 5 | 222 | 6 |
| Not known | 209 | 10 | 194 | 9 | 371 | 10 | 402 | 11 |
| Total | 2161 | 100 | 2161 | 100 | 3674 | 100 | 3674 | 100 |
|  |  |  |  |  |  |  |  |  |
| **Industrial status** |  |  |  |  |  |  |  |  |
| Non-manual | 775 | 36 | 758 | 35 | 1343 | 37 | 1258 | 34 |
| Manual | 1177 | 54 | 1209 | 56 | 1960 | 53 | 2014 | 55 |
| Unknown | 209 | 10 | 194 | 9 | 371 | 10 | 402 | 11 |
| Total | 2161 | 100 | 2161 | 100 | 3674 | 100 | 3674 | 100 |
|  |  |  |  |  |  |  |  |  |
| **Region *** |  |  |  |  |  |  |  |  |
| North | 123 | 6 | 124 | 6 | 207 | 6 | 207 | 6 |
| Yorkshire and Humberside | 194 | 9 | 194 | 9 | 323 | 9 | 322 | 9 |
| East Midlands | 166 | 8 | 159 | 7 | 258 | 7 | 270 | 7 |
| East Anglia | 69 | 3 | 71 | 3 | 123 | 3 | 125 | 3 |
| South East | 716 | 33 | 713 | 33 | 1144 | 31 | 1135 | 31 |
| South West | 150 | 7 | 153 | 7 | 313 | 9 | 306 | 8 |
| West Midlands | 202 | 9 | 203 | 9 | 366 | 10 | 365 | 10 |
| North West | 246 | 11 | 249 | 12 | 394 | 11 | 403 | 11 |
| Wales | 101 | 5 | 104 | 5 | 189 | 5 | 187 | 5 |
| Scotland | 194 | 9 | 191 | 9 | 355 | 10 | 354 | 10 |
| Not known | 0 | 0 | 0 | 0 | 2 | 0 | 0 | 0 |
| Total | 2161 | 100 | 2161 | 100 | 3674 | 100 | 3674 | 100 |

- Mother’s region of residence at the time of the child’s birth

Table B2: Numbers exposed and ORs (95% CIs) for Total Bone Tumours (ICCC3 81 to 85) by occupational exposure group

|  |  | Exposed cases | | Exposed Controls | | Informative pairs |  |  |  |  |  |  |
| --- | --- | --- | --- | --- | --- | --- | --- | --- | --- | --- | --- | --- |
| Group | | Number | % | Number | % |  | OR^a^ | 95% CI | p | OR^b^ | 95% CI | p |
| 1 | Agriculture | 39 | 2.0 | 40 | 2.0 | 71 | 1.03 | 0.65-1.64 | 0.91 | 1.02 | 0.63-1.63 | 0.94 |
| 2 | Agrochemical | 48 | 2.4 | 52 | 2.6 | 90 | 0.96 | 0.63-1.45 | 0.83 | 0.98 | 0.64-1.49 | 0.92 |
| 3 | Animals | 9 | 0.5 | 13 | 0.6 | 20 | 0.67 | 0.27-1.63 | 0.37 | 0.60 | 0.24-1.52 | 0.28 |
| 4 | Ceramics/glass | 8 | 0.4 | 9 | 0.4 | 17 | 0.89 | 0.34-2.30 | 0.81 | 0.88 | 0.34-2.27 | 0.79 |
| 5 | Coal dust | 18 | 0.9 | 18 | 0.9 | 35 | 0.94 | 0.49-1.83 | 0.87 | 0.96 | 0.49-1.86 | 0.90 |
| 6 | Construction | 139 | 7.0 | 137 | 6.8 | 235 | 1.04 | 0.81-1.35 | 0.74 | 1.07 | 0.83-1.40 | 0.59 |
| 7 | EMFs | 114 | 5.7 | 135 | 6.7 | 211 | 0.85 | 0.65-1.12 | 0.24 | 0.87 | 0.66-1.14 | 0.31 |
| 8 | Exhaust fumes | 180 | 9.0 | 156 | 7.8 | 278 | 1.21 | 0.95-1.53 | 0.12 | 1.22 | 0.96-1.55 | 0.11 |
| 9 | Fishing | 3 | 0.2 | 3 | 0.1 | 4 | 1.00 | 0.07-13.80 | 1.00 |  |  |  |
| 10 | Foodstuffs | 67 | 3.4 | 53 | 2.6 | 107 | 1.23 | 0.84-1.80 | 0.29 | 1.25 | 0.85-1.84 | 0.27 |
| 11 | Forces | 81 | 4.1 | 92 | 4.6 | 151 | 0.86 | 0.63-1.19 | 0.37 |  |  |  |
| 12 | Heat (prolonged exposure) | 59 | 3.0 | 77 | 3.8 | 117 | 0.75 | 0.52-1.08 | 0.12 | 0.77 | 0.53-1.11 | 0.16 |
| 13 | Hydrocarbons (inhaled) | 329 | 16.5 | 330 | 16.4 | 461 | 0.97 | 0.81-1.16 | 0.74 | 1.01 | 0.83-1.22 | 0.94 |
| 14 | Hydrocarbons (dermal) | 156 | 7.8 | 195 | 9.7 | 275 | **0.76** | 0.60-0.97 | <0.05 | 0.80 | 0.63-1.02 | 0.08 |
| 15 | Ionising radiation | 2 | 0.1 | 3 | 0.1 | 5 | 0.67 | 0.06-5.82 | 1.00 |  |  |  |
| 16 | Lead | 64 | 3.2 | 59 | 2.9 | 111 | 1.09 | 0.75-1.59 | 0.64 | 1.12 | 0.77-1.63 | 0.57 |
| 17 | Leather | 4 | 0.2 | 3 | 0.1 | 3 | 0.50 | 0.01-9.60 | 1.00 |  |  |  |
| 18 | Medical/Health care | 35 | 1.8 | 40 | 2.0 | 70 | 0.94 | 0.59-1.51 | 0.81 | 0.90 | 0.55-1.47 | 0.67 |
| 19 | Metal | 285 | 14.3 | 331 | 16.5 | 456 | **0.79** | 0.66-0.95 | <0.05 | **0.82** | 0.68-0.99 | <0.05 |
| 20 | Metal acid mists | 2 | 0.1 | 1 | 0.0 | 3 | 2.00 | 0.10-118.99 | 1.00 |  |  |  |
| 21 | Metal fumes | 27 | 1.4 | 39 | 1.9 | 54 | 0.74 | 0.43-1.27 | 0.28 | 0.75 | 0.44-1.29 | 0.31 |
| 22 | Metal working (oil mists) | 73 | 3.7 | 106 | 5.3 | 159 | **0.66** | 0.48-0.90 | <0.01 | **0.69** | 0.50-0.96 | <0.05 |
| 23 | Mining | 20 | 1.0 | 20 | 1.0 | 39 | 0.95 | 0.51-1.78 | 0.87 | 0.97 | 0.52-1.82 | 0.92 |
| 24 | Paints | 42 | 2.1 | 48 | 2.4 | 78 | 0.95 | 0.61-1.48 | 0.82 | 0.98 | 0.62-1.54 | 0.93 |
| 25 | Paper production |  |  |  |  | 0 |  |  |  |  |  |  |
| 26 | Plastics | 1 | 0.1 | 5 | 0.2 | 6 | 0.20 | 0.004-1.79 | 0.22 |  |  |  |
| 27 | Printing | 23 | 1.2 | 23 | 1.1 | 44 | 0.91 | 0.51-1.65 | 0.76 | 0.94 | 0.51-1.72 | 0.83 |
| 28 | Rubber | 4 | 0.2 | 3 | 0.1 | 7 | 1.33 | 0.23-9.10 | 1.00 |  |  |  |
| 29 | Social contact | 258 | 12.9 | 255 | 12.7 | 310 | 1.03 | 0.82-1.28 | 0.82 | 0.95 | 0.76-1.20 | 0.69 |
| 30 | Solvents | 68 | 3.4 | 75 | 3.7 | 110 | 0.83 | 0.57-1.21 | 0.34 | 0.85 | 0.58-1.24 | 0.40 |
| 31 | Textile dust | 37 | 1.9 | 33 | 1.6 | 62 | 1.07 | 0.65-1.76 | 0.80 | 1.10 | 0.67-1.81 | 0.72 |
| 32 | Tobacco dust | 0 | 0.0 | 1 | 0.0 | 1 |  |  |  |  |  |  |
| 33 | Wood dust | 65 | 3.3 | 58 | 2.9 | 105 | 1.19 | 0.81-1.74 | 0.38 | 1.17 | 0.79-1.73 | 0.44 |

aOR with only the implicit adjustment for the matching factors: sex, registration sub-district and period of registration

bOR additionally adjusted for paternal occupational social class (see text)

ORs in bold indicate values which differ significantly from 1 (P<0.05)

ORs in bold and underlined indicate values which differ significantly from 1 (P<0.01)

Table B3: Numbers exposed and ORs (95% CIs) for Total Soft Tissue Sarcomas (ICCC3 91 to 95) by occupational exposure group

|  |  | Exposed cases | | Exposed Controls | | Informative pairs |  |  |  |  |  |  |
| --- | --- | --- | --- | --- | --- | --- | --- | --- | --- | --- | --- | --- |
| Group | | Number | % | Number | % |  | OR^a^ | 95% CI | p | OR^b^ | 95% CI | p |
| 1 | Agriculture | 81 | 2.4 | 67 | 2.0 | 136 | 1.27 | 0.90-1.78 | 0.17 | 1.26 | 0.89-1.78 | 0.19 |
| 2 | Agrochemical | 105 | 3.1 | 99 | 2.9 | 180 | 1.07 | 0.80-1.43 | 0.66 | 1.08 | 0.80-1.46 | 0.60 |
| 3 | Animals | 22 | 0.7 | 15 | 0.4 | 29 | 1.42 | 0.68-2.97 | 0.36 | 1.35 | 0.64-2.86 | 0.43 |
| 4 | Ceramics/glass | 6 | 0.2 | 13 | 0.4 | 18 | 0.50 | 0.19-1.33 | 0.17 | 0.53 | 0.20-1.40 | 0.20 |
| 5 | Coal dust | 25 | 0.7 | 33 | 1.0 | 47 | 0.68 | 0.38-1.22 | 0.19 | 0.66 | 0.37-1.20 | 0.17 |
| 6 | Construction | 263 | 7.8 | 272 | 8.1 | 443 | 1.01 | 0.84-1.22 | 0.89 | 1.03 | 0.85-1.25 | 0.78 |
| 7 | EMFs | 185 | 5.5 | 167 | 5.0 | 293 | 1.09 | 0.87-1.37 | 0.45 | 1.10 | 0.87-1.39 | 0.42 |
| 8 | Exhaust fumes | 292 | 8.6 | 258 | 7.7 | 457 | 1.11 | 0.92-1.33 | 0.28 | 1.12 | 0.93-1.36 | 0.22 |
| 9 | Fishing | 7 | 0.2 | 6 | 0.2 | 13 | 1.17 | 0.39-3.47 | 0.78 | 1.24 | 0.41-3.68 | 0.71 |
| 10 | Foodstuffs | 122 | 3.6 | 136 | 4.0 | 224 | 0.87 | 0.67-1.13 | 0.29 | 0.88 | 0.67-1.15 | 0.36 |
| 11 | Forces | 152 | 4.5 | 158 | 4.7 | 270 | 1.00 | 0.79-1.27 | 1.00 |  |  |  |
| 12 | Heat (prolonged exposure) | 103 | 3.1 | 99 | 2.9 | 176 | 1.05 | 0.78-1.41 | 0.76 | 1.13 | 0.83-1.52 | 0.44 |
| 13 | Hydrocarbons (inhaled) | 555 | 16.4 | 544 | 16.1 | 757 | 1.01 | 0.87-1.16 | 0.91 | 1.06 | 0.91-1.23 | 0.45 |
| 14 | Hydrocarbons (dermal) | 268 | 7.9 | 285 | 8.5 | 422 | 0.94 | 0.78-1.14 | 0.56 | 0.99 | 0.81-1.21 | 0.94 |
| 15 | Ionising radiation | 2 | 0.1 | 2 | 0.1 | 4 | 1.00 | 0.07-13.80 | 1.00 |  |  |  |
| 16 | Lead | 108 | 3.2 | 120 | 3.6 | 197 | 0.89 | 0.68-1.18 | 0.43 | 0.91 | 0.68-1.20 | 0.49 |
| 17 | Leather | 11 | 0.3 | 16 | 0.5 | 25 | 0.67 | 0.30-1.48 | 0.32 | 0.72 | 0.32-1.62 | 0.42 |
| 18 | Medical/Health care | 60 | 1.8 | 65 | 1.9 | 113 | 0.88 | 0.61-1.28 | 0.51 | 0.80 | 0.55-1.17 | 0.25 |
| 19 | Metal | 513 | 15.2 | 520 | 15.4 | 761 | 1.00 | 0.87-1.16 | 0.97 | 1.02 | 0.88-1.18 | 0.81 |
| 20 | Metal acid mists | 2 | 0.1 | 5 | 0.1 | 7 | 0.40 | 0.04-2.44 | 0.45 |  |  |  |
| 21 | Metal fumes | 38 | 1.1 | 43 | 1.3 | 67 | 0.91 | 0.57-1.48 | 0.71 | 1.06 | 0.64-1.73 | 0.83 |
| 22 | Metal working (oil mists) | 135 | 4.0 | 145 | 4.3 | 245 | 0.96 | 0.75-1.23 | 0.75 | 1.00 | 0.77-1.30 | 1.00 |
| 23 | Mining | 28 | 0.8 | 36 | 1.1 | 51 | 0.65 | 0.37-1.13 | 0.13 | 0.63 | 0.36-1.12 | 0.11 |
| 24 | Paints | 57 | 1.7 | 71 | 2.1 | 109 | 0.82 | 0.56-1.19 | 0.29 | 0.86 | 0.59-1.26 | 0.44 |
| 25 | Paper production | 2 | 0.1 | 3 | 0.1 | 4 | 0.33 | 0.01-4.15 | 0.63 |  |  |  |
| 26 | Plastics | 6 | 0.2 | 6 | 0.2 | 12 | 1.00 | 0.32-3.10 | 1.00 | 1.03 | 0.33-3.19 | 0.96 |
| 27 | Printing | 28 | 0.8 | 32 | 0.9 | 55 | 0.90 | 0.53-1.52 | 0.69 | 0.94 | 0.55-1.61 | 0.83 |
| 28 | Rubber | 5 | 0.1 | 6 | 0.2 | 10 | 1.00 | 0.23-4.35 | 1.00 |  |  |  |
| 29 | Social contact | 418 | 12.4 | 416 | 12.3 | 510 | 1.00 | 0.84-1.19 | 1.00 | 0.92 | 0.77-1.11 | 0.39 |
| 30 | Solvents | 93 | 2.8 | 111 | 3.3 | 167 | 0.80 | 0.59-1.08 | 0.14 | 0.84 | 0.61-1.14 | 0.26 |
| 31 | Textile dust | 42 | 1.2 | 34 | 1.0 | 61 | 1.44 | 0.86-2.40 | 0.16 | 1.56 | 0.93-2.62 | 0.09 |
| 32 | Tobacco dust |  |  |  |  | 0 |  |  |  |  |  |  |
| 33 | Wood dust | 103 | 3.1 | 107 | 3.2 | 184 | 1.04 | 0.78-1.39 | 0.77 | 1.07 | 0.79-1.44 | 0.67 |

aOR with only the implicit adjustment for the matching factors: sex, registration sub-district and period of registration

bOR additionally adjusted for paternal occupational social class (see text)

ORs in bold indicate values which differ significantly from 1 (P<0.05)

ORs in bold and underlined indicate values which differ significantly from 1 (P<0.01)

Table B4: Numbers exposed and ORs (95% CIs) for Osteosarcoma (ICCC3 81) by occupational exposure group

|  |  | Exposed cases | | Exposed Controls | | Informative pairs |  |  |  |  |  |  |
| --- | --- | --- | --- | --- | --- | --- | --- | --- | --- | --- | --- | --- |
| Group | | Number | % | Number | % |  | OR^a^ | 95% CI | p | OR^b^ | 95% CI | p |
| 1 | Agriculture | 19 | 1.8 | 21 | 2.0 | 37 | 0.95 | 0.50-1.81 | 0.87 | 0.88 | 0.46-1.70 | 0.71 |
| 2 | Agrochemical | 23 | 2.2 | 31 | 2.9 | 49 | 0.75 | 0.43-1.32 | 0.32 | 0.74 | 0.41-1.32 | 0.31 |
| 3 | Animals | 4 | 0.4 | 7 | 0.7 | 10 | 0.43 | 0.07-1.88 | 0.34 |  |  |  |
| 4 | Ceramics/glass | 5 | 0.5 | 4 | 0.4 | 9 | 1.25 | 0.27-6.30 | 1.00 |  |  |  |
| 5 | Coal dust | 10 | 1.0 | 8 | 0.8 | 17 | 1.13 | 0.43-2.92 | 0.81 | 1.14 | 0.44-2.94 | 0.79 |
| 6 | Construction | 72 | 6.9 | 78 | 7.4 | 121 | 0.98 | 0.69-1.40 | 0.93 | 0.98 | 0.68-1.41 | 0.90 |
| 7 | EMFs | 61 | 5.9 | 68 | 6.5 | 107 | 0.88 | 0.60-1.28 | 0.50 | 0.88 | 0.60-1.29 | 0.51 |
| 8 | Exhaust fumes | 90 | 8.6 | 87 | 8.3 | 150 | 1.17 | 0.85-1.62 | 0.33 | 1.21 | 0.88-1.67 | 0.25 |
| 9 | Fishing | 0 | 0.0 | 1 | 0.1 | 1 |  |  |  |  |  |  |
| 10 | Foodstuffs | 38 | 3.7 | 29 | 2.8 | 60 | 1.31 | 0.78-2.18 | 0.30 | 1.29 | 0.77-2.19 | 0.34 |
| 11 | Forces | 39 | 3.7 | 42 | 4.0 | 68 | 0.94 | 0.59-1.52 | 0.81 |  |  |  |
| 12 | Heat (prolonged exposure) | 28 | 2.7 | 45 | 4.3 | 63 | **0.58** | 0.34-0.96 | <0.05 | **0.59** | 0.35-0.99 | <0.05 |
| 13 | Hydrocarbons (inhaled) | 170 | 16.3 | 189 | 18.0 | 256 | 0.88 | 0.69-1.13 | 0.32 | 0.93 | 0.72-1.20 | 0.59 |
| 14 | Hydrocarbons (dermal) | 77 | 7.4 | 113 | 10.7 | 144 | **0.64** | 0.46-0.89 | <0.01 | **0.66** | 0.47-0.94 | <0.05 |
| 15 | Ionising radiation | 0 | 0.0 | 2 | 0.2 | 2 |  |  |  |  |  |  |
| 16 | Lead | 41 | 3.9 | 32 | 3.0 | 66 | 1.28 | 0.78-2.07 | 0.33 | 1.33 | 0.81-2.17 | 0.26 |
| 17 | Leather | 2 | 0.2 | 1 | 0.1 | 1 |  |  |  |  |  |  |
| 18 | Medical/Health care | 17 | 1.6 | 20 | 1.9 | 35 | 0.94 | 0.49-1.83 | 0.87 | 0.91 | 0.45-1.82 | 0.79 |
| 19 | Metal | 162 | 15.6 | 184 | 17.5 | 253 | 0.83 | 0.65-1.07 | 0.15 | 0.86 | 0.67-1.10 | 0.24 |
| 20 | Metal acid mists | 1 | 0.1 | 0 | 0.0 | 1 |  |  |  |  |  |  |
| 21 | Metal fumes | 13 | 1.2 | 23 | 2.2 | 30 | 0.58 | 0.28-1.22 | 0.15 | 0.58 | 0.28-1.22 | 0.15 |
| 22 | Metal working (oil mists) | 39 | 3.7 | 61 | 5.8 | 89 | **0.62** | 0.40-0.95 | <0.05 | 0.65 | 0.42-1.00 | 0.05 |
| 23 | Mining | 11 | 1.1 | 9 | 0.9 | 19 | 1.11 | 0.45-2.73 | 0.82 | 1.13 | 0.46-2.78 | 0.80 |
| 24 | Paints | 21 | 2.0 | 27 | 2.6 | 39 | 0.95 | 0.51-1.78 | 0.87 | 0.96 | 0.50-1.83 | 0.90 |
| 25 | Paper production |  |  |  |  | 0 |  |  |  |  |  |  |
| 26 | Plastics | 1 | 0.1 | 3 | 0.3 | 4 | 0.33 | 0.01-4.15 | 0.63 |  |  |  |
| 27 | Printing | 9 | 0.9 | 13 | 1.2 | 21 | 0.62 | 0.26-1.48 | 0.28 | 0.68 | 0.28-1.65 | 0.39 |
| 28 | Rubber | 3 | 0.3 | 2 | 0.2 | 5 | 1.50 | 0.17-17.96 | 1.00 |  |  |  |
| 29 | Social contact | 136 | 13.1 | 124 | 11.8 | 152 | 1.17 | 0.85-1.61 | 0.33 | 1.12 | 0.80-1.57 | 0.50 |
| 30 | Solvents | 32 | 3.1 | 42 | 4.0 | 56 | 0.75 | 0.44-1.27 | 0.29 | 0.77 | 0.45-1.33 | 0.36 |
| 31 | Textile dust | 16 | 1.5 | 12 | 1.1 | 24 | 1.00 | 0.45-2.23 | 1.00 | 1.01 | 0.45-2.26 | 0.98 |
| 32 | Tobacco dust | 0 | 0.0 | 1 | 0.1 | 1 |  |  |  |  |  |  |
| 33 | Wood dust | 36 | 3.5 | 31 | 2.9 | 54 | 1.35 | 0.79-2.31 | 0.28 | 1.42 | 0.82-2.46 | 0.21 |

aOR with only the implicit adjustment for the matching factors: sex, registration sub-district and period of registration

bOR additionally adjusted for paternal occupational social class (see text)

ORs in bold indicate values which differ significantly from 1 (P<0.05)

ORs in bold and underlined indicate values which differ significantly from 1 (P<0.01)

Table B5: Numbers exposed and ORs (95% CIs) for Rhabdomyosarcoma (ICCC3 91) by occupational exposure group

|  |  | Exposed cases | | Exposed Controls | | Informative pairs |  |  |  |  |  |  |
| --- | --- | --- | --- | --- | --- | --- | --- | --- | --- | --- | --- | --- |
| Group | | Number | % | Number | % |  | OR^a^ | 95% CI | p | OR^b^ | 95% CI | p |
| 1 | Agriculture | 42 | 2.2 | 30 | 1.6 | 66 | 1.54 | 0.94-2.52 | 0.09 | 1.55 | 0.94-2.56 | 0.09 |
| 2 | Agrochemical | 55 | 2.9 | 54 | 2.8 | 95 | 1.07 | 0.71-1.59 | 0.76 | 1.10 | 0.73-1.65 | 0.66 |
| 3 | Animals | 16 | 0.8 | 14 | 0.7 | 23 | 1.09 | 0.48-2.47 | 0.84 | 1.10 | 0.49-2.49 | 0.82 |
| 4 | Ceramics/glass | 2 | 0.1 | 6 | 0.3 | 8 | 0.33 | 0.03-1.86 | 0.29 |  |  |  |
| 5 | Coal dust | 15 | 0.8 | 15 | 0.8 | 22 | 1.00 | 0.43-2.31 | 1.00 | 1.02 | 0.44-2.36 | 0.96 |
| 6 | Construction | 148 | 7.7 | 160 | 8.3 | 245 | 1.02 | 0.80-1.32 | 0.85 | 1.01 | 0.78-1.31 | 0.94 |
| 7 | EMFs | 120 | 6.2 | 80 | 4.2 | 168 | **1.67** | 1.22-2.28 | <0.01 | **1.63** | 1.19-2.24 | <0.01 |
| 8 | Exhaust fumes | 173 | 9.0 | 142 | 7.4 | 259 | 1.25 | 0.98-1.60 | 0.07 | 1.24 | 0.97-1.60 | 0.09 |
| 9 | Fishing | 2 | 0.1 | 1 | 0.1 | 3 | 2.00 | 0.10-117.99 | 1.00 |  |  |  |
| 10 | Foodstuffs | 66 | 3.4 | 74 | 3.8 | 118 | 0.82 | 0.57-1.17 | 0.27 | 0.89 | 0.61-1.29 | 0.54 |
| 11 | Forces | 88 | 4.6 | 84 | 4.4 | 153 | 1.13 | 0.82-1.55 | 0.47 |  |  |  |
| 12 | Heat (prolonged exposure) | 56 | 2.9 | 45 | 2.3 | 90 | 1.31 | 0.86-1.98 | 0.21 | 1.43 | 0.93-2.20 | 0.10 |
| 13 | Hydrocarbons (inhaled) | 314 | 16.3 | 298 | 15.5 | 429 | 1.12 | 0.93-1.36 | 0.23 | 1.17 | 0.96-1.43 | 0.11 |
| 14 | Hydrocarbons (dermal) | 152 | 7.9 | 161 | 8.4 | 240 | 0.95 | 0.74-1.23 | 0.70 | 0.96 | 0.74-1.25 | 0.77 |
| 15 | Ionising radiation | 1 | 0.1 | 0 | 0.0 | 1 |  |  |  |  |  |  |
| 16 | Lead | 65 | 3.4 | 74 | 3.8 | 118 | 0.87 | 0.61-1.25 | 0.46 | 0.87 | 0.61-1.26 | 0.47 |
| 17 | Leather | 7 | 0.4 | 12 | 0.6 | 17 | 0.55 | 0.20-1.47 | 0.23 | 0.60 | 0.22-1.65 | 0.32 |
| 18 | Medical/Health care | 31 | 1.6 | 38 | 2.0 | 63 | 0.80 | 0.49-1.31 | 0.38 | 0.73 | 0.44-1.22 | 0.23 |
| 19 | Metal | 300 | 15.6 | 304 | 15.8 | 445 | 0.99 | 0.82-1.19 | 0.89 | 0.98 | 0.81-1.19 | 0.84 |
| 20 | Metal acid mists | 2 | 0.1 | 2 | 0.1 | 4 | 1.00 | 0.07-13.80 | 1.00 |  |  |  |
| 21 | Metal fumes | 23 | 1.2 | 18 | 0.9 | 33 | 1.54 | 0.77-3.09 | 0.23 | 1.83 | 0.88-3.83 | 0.11 |
| 22 | Metal working (oil mists) | 78 | 4.1 | 85 | 4.4 | 143 | 0.99 | 0.71-1.37 | 0.93 | 0.97 | 0.69-1.36 | 0.86 |
| 23 | Mining | 18 | 0.9 | 15 | 0.8 | 23 | 1.09 | 0.48-2.47 | 0.84 | 1.12 | 0.49-2.53 | 0.79 |
| 24 | Paints | 31 | 1.6 | 40 | 2.1 | 59 | 0.74 | 0.44-1.23 | 0.24 | 0.79 | 0.47-1.34 | 0.39 |
| 25 | Paper production | 0 | 0.0 | 1 | 0.1 | 1 |  |  |  |  |  |  |
| 26 | Plastics | 3 | 0.2 | 5 | 0.3 | 8 | 0.60 | 0.09-3.08 | 0.73 |  |  |  |
| 27 | Printing | 17 | 0.9 | 20 | 1.0 | 32 | 0.88 | 0.44-1.77 | 0.72 | 0.89 | 0.44-1.78 | 0.74 |
| 28 | Rubber | 2 | 0.1 | 3 | 0.2 | 5 | 0.67 | 0.06-5.82 | 1.00 |  |  |  |
| 29 | Social contact | 231 | 12.0 | 225 | 11.7 | 275 | 0.95 | 0.75-1.20 | 0.67 | 0.88 | 0.69-1.13 | 0.31 |
| 30 | Solvents | 51 | 2.7 | 65 | 3.4 | 91 | 0.75 | 0.50-1.14 | 0.17 | 0.79 | 0.52-1.20 | 0.26 |
| 31 | Textile dust | 26 | 1.4 | 20 | 1.0 | 37 | 1.31 | 0.68-2.52 | 0.41 | 1.44 | 0.74-2.81 | 0.28 |
| 32 | Tobacco dust |  |  |  |  | 0 |  |  |  |  |  |  |
| 33 | Wood dust | 60 | 3.1 | 64 | 3.3 | 112 | 1.00 | 0.69-1.45 | 1.00 | 1.01 | 0.69-1.48 | 0.97 |

aOR with only the implicit adjustment for the matching factors: sex, registration sub-district and period of registration

bOR additionally adjusted for paternal occupational social class (see text)

ORs in bold indicate values which differ significantly from 1 (P<0.05)

ORs in bold and underlined indicate values which differ significantly from 1 (P<0.01)

Table B6 Paternal Occupational Exposures and ORs for Ewing Sarcoma Family of Tumours

|  |  | Exposed cases | | Exposed Controls | | Informative pairs |  |  |  |  |  |  |
| --- | --- | --- | --- | --- | --- | --- | --- | --- | --- | --- | --- | --- |
| Group | | Number | % | Number | % |  | OR^a^ | 95% CI | p | OR^b^ | 95% CI | p |
| 1 | Agriculture | 22 | 1.9 | 25 | 2.2 | 40 | 0.90 | 0.49-1.68 | 0.75 | 0.95 | 0.51-1.78 | 0.87 |
| 2 | Agrochemical | 30 | 2.7 | 28 | 2.5 | 50 | 1.08 | 0.62-1.89 | 0.78 | 1.15 | 0.65-2.01 | 0.63 |
| 3 | Animals | 6 | 0.5 | 5 | 0.4 | 10 | 1.00 | 0.23-4.35 | 1.00 |  |  |  |
| 4 | Ceramics/glass | 2 | 0.2 | 6 | 0.5 | 8 | 0.33 | 0.03-1.86 | 0.29 |  |  |  |
| 5 | Coal dust | 8 | 0.7 | 12 | 1.1 | 20 | 0.67 | 0.27-1.63 | 0.37 | 0.67 | 0.28-1.65 | 0.39 |
| 6 | Construction | 85 | 7.5 | 81 | 7.2 | 149 | 1.01 | 0.74-1.40 | 0.94 | 1.06 | 0.76-1.47 | 0.74 |
| 7 | EMFs | 63 | 5.6 | 77 | 6.8 | 118 | 0.84 | 0.59-1.21 | 0.36 | 0.86 | 0.60-1.24 | 0.42 |
| 8 | Exhaust fumes | 98 | 8.7 | 87 | 7.7 | 154 | 1.05 | 0.77-1.44 | 0.75 | 1.06 | 0.76-1.46 | 0.74 |
| 9 | Fishing | 3 | 0.3 | 2 | 0.2 | 3 | 2.00 | 0.10-117.99 | 1.00 |  |  |  |
| 10 | Foodstuffs | 38 | 3.4 | 34 | 3.0 | 65 | 1.03 | 0.63-1.68 | 0.90 | 1.06 | 0.65-1.73 | 0.81 |
| 11 | Forces | 47 | 4.2 | 60 | 5.3 | 90 | 0.76 | 0.50-1.16 | 0.21 |  |  |  |
| 12 | Heat (prolonged exposure) | 33 | 2.9 | 40 | 3.5 | 61 | 0.85 | 0.51-1.40 | 0.52 | 0.83 | 0.50-1.39 | 0.49 |
| 13 | Hydrocarbons (inhaled) | 190 | 16.8 | 177 | 15.7 | 246 | 1.02 | 0.79-1.30 | 0.90 | 1.02 | 0.79-1.32 | 0.90 |
| 14 | Hydrocarbons (dermal) | 94 | 8.3 | 103 | 9.1 | 152 | 0.85 | 0.62-1.17 | 0.33 | 0.90 | 0.65-1.25 | 0.53 |
| 15 | Ionising radiation | 1 | 0.1 | 2 | 0.2 | 3 | 0.50 | 0.01-9.60 | 1.00 |  |  |  |
| 16 | Lead | 29 | 2.6 | 33 | 2.9 | 56 | 0.87 | 0.51-1.47 | 0.59 | 0.86 | 0.51-1.47 | 0.58 |
| 17 | Leather | 2 | 0.2 | 2 | 0.2 | 3 | 2.00 | 0.10-117.99 | 1.00 |  |  |  |
| 18 | Medical/Health care | 23 | 2.0 | 24 | 2.1 | 42 | 1.00 | 0.55-1.83 | 1.00 | 0.93 | 0.50-1.72 | 0.82 |
| 19 | Metal | 151 | 13.4 | 173 | 15.3 | 242 | 0.79 | 0.62-1.02 | 0.07 | 0.81 | 0.62-1.05 | 0.11 |
| 20 | Metal acid mists | 1 | 0.1 | 2 | 0.2 | 3 | 0.50 | 0.01-9.60 | 1.00 |  |  |  |
| 21 | Metal fumes | 13 | 1.1 | 19 | 1.7 | 25 | 0.79 | 0.36-1.73 | 0.55 | 0.81 | 0.37-1.79 | 0.61 |
| 22 | Metal working (oil mists) | 45 | 4.0 | 53 | 4.7 | 81 | 0.76 | 0.49-1.18 | 0.22 | 0.80 | 0.51-1.25 | 0.32 |
| 23 | Mining | 9 | 0.8 | 13 | 1.2 | 22 | 0.69 | 0.30-1.62 | 0.40 | 0.70 | 0.30-1.64 | 0.41 |
| 24 | Paints | 21 | 1.9 | 24 | 2.1 | 40 | 0.82 | 0.44-1.53 | 0.53 | 0.84 | 0.45-1.57 | 0.59 |
| 25 | Paper production |  |  |  |  | 0 |  |  |  |  |  |  |
| 26 | Plastics | 0 | 0.0 | 2 | 0.2 | 2 |  |  |  |  |  |  |
| 27 | Printing | 17 | 1.5 | 10 | 0.9 | 26 | 1.60 | 0.73-3.53 | 0.24 | 1.55 | 0.70-3.46 | 0.28 |
| 28 | Rubber | 2 | 0.2 | 1 | 0.1 | 3 | 2.00 | 0.10-117.99 | 1.00 |  |  |  |
| 29 | Social contact | 134 | 11.8 | 159 | 14.1 | 182 | 0.92 | 0.68-1.22 | 0.55 | 0.84 | 0.62-1.14 | 0.27 |
| 30 | Solvents | 40 | 3.5 | 38 | 3.4 | 62 | 0.88 | 0.53-1.45 | 0.61 | 0.86 | 0.52-1.43 | 0.55 |
| 31 | Textile dust | 29 | 2.6 | 19 | 1.7 | 41 | **1.93** | 1.01-3.68 | <0.05 | **1.99** | 1.04-3.79 | <0.05 |
| 32 | Tobacco dust |  |  |  |  | 0 |  |  |  |  |  |  |
| 33 | Wood dust | 37 | 3.3 | 31 | 2.7 | 58 | 1.15 | 0.69-1.92 | 0.60 | 1.09 | 0.64-1.84 | 0.75 |

aOR with only the implicit adjustment for the matching factors: sex, registration sub-district and period of registration

bOR additionally adjusted for paternal occupational social class (see text)

ORs in bold indicate values which differ significantly from 1 (P<0.05)

ORs in bold and underlined indicate values which differ significantly from 1 (P<0.01)
